# CPEB4 Knockout Mice Exhibit Normal Hippocampus-Related Synaptic Plasticity and Memory

**DOI:** 10.1371/journal.pone.0084978

**Published:** 2013-12-30

**Authors:** Li-Yun Tsai, Yu-Wei Chang, Pei-Yi Lin, Hsin-Jung Chou, Ta-Jen Liu, Ping-Tao Lee, Wen-Hsuan Huang, Yueh-Liang Tsou, Yi-Shuian Huang

**Affiliations:** 1 Institute of Biomedical Sciences, Academia Sinica, Taipei, Taiwan; 2 Graduate Institute of Life Sciences, National Defense Medical Center, Taipei, Taiwan; 3 Institute of Neuroscience, National Yang-Ming University, Taipei, Taiwan; Western University of Health Sciences, United States of America

## Abstract

Regulated RNA translation is critical to provide proteins needed to maintain persistent modification of synaptic strength, which underlies the molecular basis of long-term memory (LTM). Cytoplasmic polyadenylation element-binding proteins (CPEBs) are sequence-specific RNA-binding proteins and regulate translation in various tissues. All four CPEBs in vertebrates are expressed in the brain, including the hippocampal neurons, suggesting their potential roles in translation-dependent plasticity and memory. Although CPEB1 and CPEB3 have been shown to control specific kinds of hippocampus-related LTM, the role of CPEB2 and CPEB4 in learning and memory remains elusive. Thus, we generated CPEB4 knockout (KO) mice and analyzed them using several behavioral tests. No difference was found in the anxiety level, motor coordination, hippocampus-dependent learning and memory between the KO mice and their wild-type (WT) littermates. Electrophysiological recordings of multiple forms of synaptic plasticity in the Schaffer collateral pathway-CA1 neurons also showed normal responses in the KO hippocampal slices. Morphological analyses revealed that the CPEB4-lacking pyramidal neurons possessed slightly elongated dendritic spines. Unlike its related family members, CPEB1 and CPEB3, CPEB4 seems to be dispensable for hippocampus-dependent plasticity, learning and memory.

## Introduction

The CPEB family of RNA-binding proteins in vertebrates contains CPEB1, CPEB2, CPEB3 and CPEB4, all of which are expressed in the brain [1] and share structure and sequence identity in the C-terminal RNA-binding domain (RBD) [2]. CPEB2, CPEB3 and CPEB4 are more closely related to each other than they are to CPEB1. For example, CPEBs2–4 are of 96% and ∼25–35% identity in the RBD and the N-terminal regulatory region, respectively, but they share 45% identity with CPEB1 only in the RBD region [2]. Although CPEB1-controlled translation is characterized at mechanistic details in the model organism, *Xenopus* oocytes, it is involved in a wild range of physiological functions, including germ cell development, cell cycle progression, cellular senesce, metabolism and memory [3], [4], [5], [6], [7], [8], [9]. In contrast, the molecular actions of how CPEBs2–4 control translation have been recently uncovered by several laboratories. CPEB2 and CPEB3 can interact with eukaryotic elongation factor 2 (eEF2), to downregulate eEF2’s GTPase activity and hence slow down protein synthesis at elongation [10]. Activation of N-methyl-D-aspartate (NMDA) receptors in neurons triggers calpain 2-mediated proteolysis of the repressor CPEB3, leading to translational upregulation of CPEB3′s target RNAs in a polyadenylation-independent manner [2], [11]. Alternatively, monoubiquitination of CPEB3 by the E3 ligase, neuralized1, converts CPEB3 from a repressor to an activator to promote polyadenylation-induced translation of GluA1 and GluA2 RNAs [12]. In terms of CPEB4, several studies have demonstrated its function in both mitotic and meiotic cell cycle progression using knockdown or overexpression approaches [8], [13], [14]. CPEB4 was identified as an RORγt-transcribed gene during T cell development. Overexpression of CPEB4 in CD4+/CD8+ thymocytes increased the percentage of thymocytes arrested at G1 stage [14], suggesting that CPEB4-inhibited proliferation likely facilitates gene rearrangement and maturation of T cells. Moreover, CPEB4 can promote polyadenylation and translation of RNAs required for second meiotic division in *Xenopus* oocytes and G2/M mitotic entry in HeLa cells [8], [13]. Most interestingly, CPEB4 is up-expressed in pancreatic ductal adenocarcinomas (PDA) and glioblastomas. The elevated CPEB4 expression in PDA increases polyadenylation-induced translation of tissue plasminogen activator mRNA, a key contributor to PDA malignancy [15]. Knocking down CPEB4 in pancreatic cancer cells, RWP-1 and Capan-1, reduced tumorigenic properties (i.e. tumor growth, vascularization and invasion) of those cells in nude mice [15]. Despite the fact that CPEB4 and ubiquitin-modified CPEB3 can promote polyadenylation-induced translation of their target RNAs, the molecular mechanisms by which they do so are largely unrevealed.

In addition to its role in cell cycle, CPEB4 is also expressed in post-mitotic neurons where it supports neuronal viability, because CPEB4 knockdown neurons had poorer survival rate [16]. CPEB4 shuttles between nucleocytoplasmic compartments with longer retention in the cytoplasm and becomes nuclear in response to focal ischemia *in vivo* and when stimulated neurons with NMDA [16], yet the physiological function of nuclear CPEB4 remains unidentified. To investigate whether the ablation of *cpeb4* gene *in vivo* can recapitulate any defect identified previously using the knockdown approach or influence learning and memory, we generated the CPEB4 KO mice. The growth and fertility of CPEB4 null mice appeared overtly normal. The anxiety level and motor coordination of the KO mice as determined by open field, elevated plus maze (EPM) and rotarod tests were comparable to their WT littermates. Morris water maze and contextual fear conditioning tests indicated that hippocampus-dependent learning and memory ability of the CPEB4 null mice was normal. Multiple forms of synaptic plasticity, such as long-term potentiation (LTP) and long-term depression (LTD) in the Schaffer collateral (SC) pathway-CA1 neurons showed no abnormalities in the KO hippocampal slices. Even though the dendritic spines of the KO neurons were slightly lengthened, this subtle disparity was not sufficient to cause obvious abnormalities in hippocampus-related plasticity and memory.

## Materials and Methods

### Antibodies and Chemicals

Antibodies used in the study included the following: LRP130 (cat No. sc-66845) and GFP (cat No. 29779) antibodies were from Santa Cruz Biotechnology and AnaSpec, respectively. The CPEB4 polyclonal antibody was raised using the N-terminal 427 amino acids (a.a.) of rat CPEB4 produced in *Escherichia coli*. With the exception of the Vectastain Elite ABC kit (cat No. PK-6102, Vector labs), all of the other chemicals were purchased from Sigma-Aldrich.

### Ethics Statement

All works involving animals in this study have been approved by Institutional Animal Care and Use Committee (IACUC) of Academia Sinica and compliant with Taiwan National Science Council guidelines for ethical treatment of animals.

### Animals

All of the experimental protocols were performed in accordance with the guidelines of the IACUC. The mice were housed under a 12-h light/dark cycle in a climate-controlled room with *ad libitum* access to food and water. All efforts were made to minimize the number of animals used and their suffering. The WT and KO male mice used in this study were littermates from heterozygous matings. Once the genotype of the mice was determined, the WT and KO males were housed 4–5 per cage after weaning.

### Construction of the CPEB4 Targeting Vector and Production of Knockout Mice

The genomic BAC clone (RP23-106P18) containing the entire C57BL/6J mouse *cpeb4* gene was obtained from Invitrogen and used for constructing the targeting vector by the recombineering technique following the manufacturer’s protocols (Gene Bridges). The exon 2 along with its flanking upstream ∼9.5-kb intron 1 and downstream ∼7.2-kb intron 2 was retrieved from the BAC clone to the PL253 vector by homologous recombination. This PL253 plasmid contains a *Mc1*-driven thymidine kinase cassette for negative selection in embryonic stem (ES) cells. A loxP-PGK-Neo-loxP cassette amplified from the plasmid PL452 was first recombined into the 5′ end of exon 2 and excised with Cre recombinase (New England Biolab) *in vitro* to result in a single loxP site. The resulting plasmid was recombineered with the Frt-PGK-Neo-Frt-loxP cassette to the 3′ end of exon 2. The NotI-linearized targeting construct was electroporated into C57BL/6J ES cells. The genomic DNA was subsequently isolated from G418 (positive selection) and gancyclovir (negative selection)-resistant clones. Two clones out of 25 G418-resistant clones with correct targeting were amplified and injected into c2J blastocysts [17]. Both clones derived germline-transmitted lines, which were then crossed with C57BL6 protamine-Cre mice. The male progenies carried the targeted allele and protamine-Cre transgene were crossed with C57BL6 female mice to remove the exon 2 in the sperm to derive global heterozygous KO mice. The resulting litters were selected for deletion of the exon 2 by Southern blotting.

### Total RNA Extraction, Random Primer Labeling, RT-PCR and Quantitative PCR (qPCR)

Total RNA was extracted using Trizol reagent (Invitrogen). Thirty µg total RNAs isolated from WT, heterozygous and KO brains were used for Northern blotting. The 632-bp BglII-NcoI DNA fragment excised from the exon 1 of mCPEB4 cDNA was used to synthesize the radiolabeled probe for Northern blotting by random primer labeling. The other probes for Southern blotting were synthesized in the same way using the DNA templates amplified from the mouse genome with the primers: 5′-end probe, 5′-GCAGGTAAGAGAACCTATAACCAAG-3′ and 5′-TCCTACCATGTCTAGGAGTTC-3′; 3′-end probe, 5′-TTACACTGCCACCTTAAGT-3′ and 5′-CAGGTCCTCATGCTTCTG-3′; and H3 KO probe, 5′-TGGATGCCACCTCATACATG-3′ and 5′-TTGCTTCCCAGAAACATAACC-3′. The cDNAs were reverse transcribed from WT and KO brain RNAs using oligo-dT and ImPromII Reverse Transcriptase (Promega), followed by PCR or qPCR analyses. The primer sets for PCR were: the sense primer exon1, 5′-CGCAGAGGGCTGAACGGTGG-3′ and the antisense primer exon8, 5′-AGGGCCGGATCTGCACTGGT-3′; the sense primer exon7, 5′-CCAACCATCAAGGATAAACCA-3′ and the antisense primer exon9, 5′-AACAAAGCGGGCACTGATAG-3′. Quantitative PCR was performed using the Universal Probe Library and Lightcycler 480 system (Roche). The data analysis was performed using the comparative *C_t_* (threshold cycle value) method with β-actin mRNA as the reference. The PCR primers that were used included the following: CPEB4, 5′-CCAACCATCAAGGATAAACCA-3′ and 5′-AGCCATCCATCACAAAGTCA-3′; β-actin, 5′-AAGGCCAACCGTGAAAAGAT-3′ and 5′-GTGGTACGACCAGAGGCATAC-3′.

### Genotyping of CPEB4 Embryos and Mice

To obtain genotyping within 2 h for WT and KO neuron cultures, we routinely determined the genotypes of the CPEB4 mutant embryos and mice by PCR using tail biopsies and the KAPA mouse genotyping kit (KK7302, KAPA Biosystems) according to the manufacturer’s protocol. Briefly, tail samples were lysed in 20 µl of KAPA extract buffer for 20 min at 75°C and then 5 min at 95–100°C. The DNA sample was then diluted with 60 µl H_2_O, and 0.5 µl of DNA sample was used for a 10-µl PCR reaction. The sense primer, CP4F1, 5′-CTCTCATTCACTTGACTGAC-3′ and two antisense primers CP4R1, 5′-CAGAACATACTCTAGCACGT-3′ and CP4R2, 5′-TGTATCAGTGATATGACATGC-3′ at a 2∶1∶1 ratio were used to amplify the WT and KO alleles, respectively. The three primers were then used in a multiplex PCR with KAPA2G fast PCR genotyping mix under the following amplification conditions: 95°C for 3 min, 33 cycles of 95°C for 15 s, 55°C for 15 s, 72°C for 15 s and 2 min incubation at 72°C at the end of the run. The amplified products were resolved on a 1% agarose gel.

### Primary Neuronal Cultures and DNA Transfection

The cortices and hippocampi of E18 embryos from heterozygous matings were isolated and kept individually in Hank’s balanced salt solution (HBSS) on ice; meanwhile the tails were collected for genotyping. Once the embryos’ genotypes were determined, the WT and KO cerebral cortices were pooled and digested in papain solution (0.6 mg/ml papain and DNase I, 0.5 mM ethylenediaminetetraacetic acid (EDTA), 0.2 mg/ml cysteine and 1.5 mM CaCl_2_ in HBSS). The dissociated WT and KO neurons were cultured in Neurobasal medium with B27 supplement. Neurons were plated on poly-L-lysine coated 18-mm coverslips in a 12-well plate with the density of 3×10^5^ cells/well. Delivery of DNA into the neurons was carried out using calcium phosphate transfection as previously described [18]. Neurons at 14 days *in vitro* (DIV) transfected with the enhanced green fluorescent protein (EGFP) plasmid were fixed on DIV18 prior to immunostaining.

### Immunohistochemistry, Immunofluorescence Staining, Imaging Acquisition and Quantification

Coronal sections of WT and KO brains after 10 min fixation in 4% formaldehyde and 20 min antigen retrieval in 10 mM sodium citrate buffer, pH 6 at 70°C, were washed twice with Tris-buffered saline (TBS) and permeabilized with 0.2% TritonX-100 in TBS. After three washes with TBS and 1 h blocking in 10% horse serum, the slices were incubated with affinity-purified CPEB4 antibody at 4°C overnight. The slices were rinsed with TBS three times, and then incubated with biotinylated anti-rabbit IgG at room temperature (RT) for 1 h, followed by three washes with TBS and the addition of avidin-biotin complex at RT for 30 min. After three washes with TBS, the slices were incubated with the 3, 3′-diaminobenzidine (DAB) substrate until the appearance of brownish color and then mounted on to slides for image acquisition. Cultured neurons transfected with the EGFP plasmid were processed for immunofluorescence staining as previously described [18]. The fluorescence images were acquired using a LSM 510 META confocal microscope (Carl Zeiss) with a Plan-Apochromat 40X oil objective lens. Each image (2048×2048 pixels) consisted of a stack of 7 to 9 Z-series images at 0.5 µm spacing. The images were quantified using the MetaMorph software and then exported into Excel and GraphPad Prism for the analyses. Approximately 3000 spines within 20 µm dendritic segments 30 µm away from the somas of 30 neurons were measured in each group. The statistical significance was determined using Student’s *t*-test.

### Behavior Assays

All male mice used were 2–3 months old. All behavioral studies were performed by an observer who did not know the genotype of the mice until the entire test had been completed and conducted during the light phase (between 13∶30–18∶00) of the light/dark cycle (lights on 8∶00–20∶00). Open field: The mice were individually placed in the apparatus consisting of 4 transparent Plexiglas arenas (480×480×350 mm) in which the floor and walls were covered with black paper to prevent any interference from the movements of the neighboring mouse. Each mouse was released into a corner of the box and allowed to explore for 60 min. The total travel distance, path length and percentage of time spent in the center were recorded during first 10 min as well as the subsequent 50 min, which were analyzed separately using the TopScan system (Clever Sys.). Elevated plus maze (EPM): The EPM consisted of two open arms (30 cm×5 cm) with a 1 cm (in height) ledge and two enclosed arms with 15 cm (in height) walls. The arms, central square and walls were made of white plastic plates. The maze was elevated to a height of 50 cm above the floor during the task in which the mice were placed in the central area of the maze, facing one of the open arms. The mouse behaviors were recorded in a 5-min testing period, and the percentage of time spent on the open arm by the mouse was analyzed using the TopScan system. Rotarod test: The ROTA-ROD MK660D machine was used in this test. The mice were placed on rotating drums with gradually accelerated speeds up to 44 rpm. The time each mouse was able to maintain its balance on the rotating rod at a specific speed was measured (Irvine et al. 2011). Morris water maze [19]: The mice were trained in a circular pool with a diameter of 1.54 m, filled with milky water, which was maintained at 20°C. A circular platform (13 cm in diameter and 12.5 cm in height) was placed in the center of one quadrant (target quadrant) and hidden 1 cm beneath the water surface. Training for the hidden platform version of the Morris water maze consisted of four trials each day for four consecutive days. The probe trial was administered 24 h after the four training days. The percentage of time spent by the mouse in each quadrant was recorded. Lastly, the escape platform, which was marked by a visible flag, was placed to ensure the swimming ability and visual acuity of the mice. For all of the trials, the maximal swimming duration was 60 sec and the inter-trial interval (ITI) was 15 min. The trajectories of the mice were recorded and analyzed using the video tracking and measuring system, TrackMot (Singa Technology Corporation, Taiwan). Contextual fear conditioning: On the day of fear conditioning, the mice were placed in the chamber, and a 2 sec 0.5 mA foot shock was given every 2 min, four times. Two minutes after the final shock, the mice were then removed and placed back into their cages. Extinction trials began 24 h later and were performed in the same chamber. Each trial duration was 6 min and the percentage of freezing was analyzed in the acquisition and extinction training periods. The extinction memory recall was conducted a week after the extinction training. All of the behavioral data were expressed as the mean ± standard error of the mean (SEM) and analyzed using STATISTICA software. For the statistical analysis, Student’s t-test, one-way analysis of variance (ANOVA) and two-way ANOVA with repeated measures were used to determine the differences. Values of *P*<0.05 were considered to be statistically significant.

### Slice Preparation and Field Recording

Either WT or KO male mouse obtained from the heterozygous mating was decapitated and the brain was immediately isolated and placed in ice-cold artificial cerebral spinal fluid (aCSF, 124 mM NaCl, 4.4 mM KCl, 1 mM NaH_2_PO_4_, 1.3 mM MgSO_4_, 10 mM D-glucose, 26 mM NaHCO_3_, 2.5 mM CaCl_2_, and 0.5 mM ascorbic acid, pH 7.4) and oxygenated with 95% O_2_ and 5% CO_2_. Transverse hippocampal slices (400 µm thick) were prepared using a microslicer (DTK-1000, DSK, Japan) and recovered in a submerged holding chamber perfused with oxygenated aCSF at 28°C for at least 2 h. The slices were then transferred to an immersion-type chamber perfused with aCSF at a flow rate of 2–3 ml/min and maintained at 30±1°C to record the field excitatory postsynaptic potential (fEPSP) in the SC pathway. A concentric bipolar tungsten stimulating electrode (teflon-coated tungsten wire, 0.05 mm bare, 0.1 mm coated, AM79550) was placed in the stratum radiatum near the CA2 region and a glass recording microelectrode (thin-wall borosilicate capillary glass with microfilament, AM615500, A-M Systems) filled with aCSF (resistance of 2–5 MΩ) was placed in the stratum radiatum of the CA1 region. The input-output responses were measured using the stimulus intensity from 20 to 110 µA. Paired-pulse facilitation (PPF) was measured at interpulse intervals (IPIs) of 10, 30, 50, 100, 150, 200, and 250 msec. Baseline stimulation (0.017 Hz, 0.1 ms pulse duration, biphasic) was adjusted to evoke 30–40% and 50% of the maximal response for LTP and LTD, respectively, using the stimulator (Model 2100, A-M Systems, USA). The quantification of synaptic transmission strength was measured using the slope of the fEPSP (using the minimum slope from 10–90% of the rising phase). A stable baseline was acquired 20–30 min before stimulation. LTD was induced by low frequency stimulation (LFS): 1 Hz 15-min stimulation (900 stimuli) or paired-pulse LFS (PP-LFS, 50 ms IPI), in slices from 3–4-week-old juvenile mice. LTP was evoked by high frequency stimulation (HFS): one train of 100 Hz, four trains of 100 Hz (5-min ITI), one train of theta burst stimulation (TBS, nine bursts of four pulses at 100 Hz, 200-msec interburst interval) or four trains of TBS (5-min ITI) in slices from 3-month-old adult mice. The average fEPSP slope measured at the indicated time after stimulation was used for statistical comparisons using Student’s *t*-test.

## Results

### Generation of CPEB4 KO Mice

**Figure 1:**
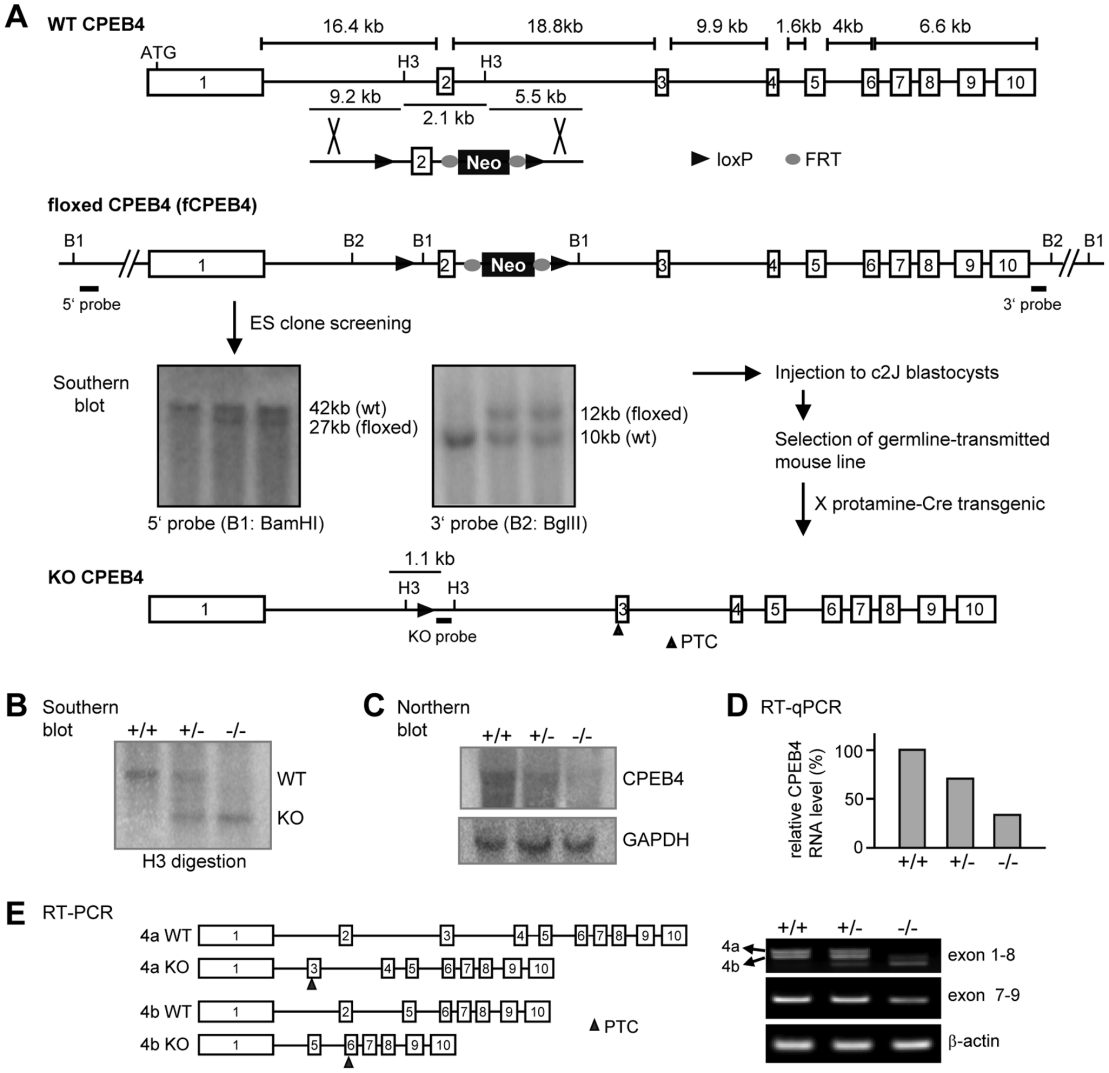
Generation and characterization of CPEB4 null mice. (**A**) Schematic illustration of the targeting strategy. The *cpeb4* gene consists of 10 exons (numbered boxes) and spans a region of approximately 60-kb (WT, wild-type). The targeting vector containing the flip recombinase target (FRT)-flanked neomycin-resistance gene (Neo) and loxP-flanked exon 2 cassette was inserted into the *cpeb4* gene by homologous recombination. The correctly targeted locus was examined by Southern blotting using DNA digested with BamHI (B1) or BglII (B2) and probed with a 5′-end or 3′-end probe, respectively. The mouse line carrying the floxed allele of CPEB4 (fCPEB4) was then crossed with a transgenic male mouse expressing Cre recombinase in the sperm (protamine-Cre) to produce the knockout (KO) allele. (**B**) The WT and KO alleles were distinguished on a Southern blot using DNA digested with HindIII (H3). The brain RNAs isolated from male offspring carrying WT (+/+), heterozygous (+/−) or KO (−/−) alleles were analyzed by (**C**) Northern blotting using a probe against exon 1 of CPEB4 RNA or GAPDH RNA (loading control). (**D**) RT-qPCR analysis. The brain RNAs were reverse transcribed and PCR-amplified for CPEB4 and β-actin. The CPEB4 RNA level after normalization with the β-actin signal was expressed as a relative ratio to the WT which was arbitrarily set to 100%. (**E**) RT-PCR confirmed the presence of exon 2-deleted CPEB4 transcripts in the KO brain. The doublet bands amplified from the WT or KO cDNA were resulted from the presence of two alternatively spliced isoforms (4a and 4b). PTC, premature termination codon.

**Figure 2:**
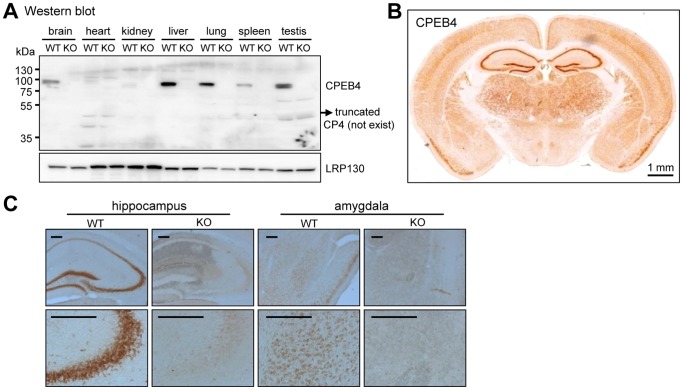
No CPEB4 protein was detected in the KO tissues. (**A**) Western blot analysis. Expression of CPEB4 in the WT and KO tissues was detected using affinity-purified polyclonal CPEB4 antibody. Equal transfer and loading were determined with LRP130 antibody. (**B**) Distribution of CPEB4 in subregions of the brain was revealed by immunohistochemical staining of coronal sections using the same affinity-purified CPEB4 antibody. (**C**) High magnification of CPEB4 images were shown in the hippocampus and amygdala areas. Both assays showed no immunodetectable signals in the KO tissues. Scale, 0.2 mm unless specified.

To investigate the role of CPEB4 in learning and memory, the Cre-loxP strategy was used to generate CPEB4 global KO mice in a C57BL/6 genetic background (Figure 1A). The linearized targeting vector containing exon 2 (82-bp) of the *cpeb4* gene and the phosphoglycerate kinase (PGK) promoter-driven neomycin (Neo) selection marker flanked by two loxP sites followed by a thymidine kinase cassette for negative selection, was electroporated into C57BL/6 ES cells. The genomic DNAs isolated from G418 and gancyclovir-resistant clones were digested with BamHI or BglII and analyzed on a Southern blot with a 5′-end or 3′-end probe flanking the targeted locus, respectively (Figure 1A). Two correctly targeted clones were amplified and injected into C57BL/6-Tyr^c-2J^ (c2J) blastocysts to generate chimeric mice. Because c2J mice are C57BL/6 albino mice, they have the BL/6 genetic background except carrying a mutation in the tyrosinase gene [17]. Germline transmission was examined only in the progenies of black coat color obtained by breeding the chimeric males with c2J females since the black fur offspring were derived from the targeted BL/6 ES cells. The germline-transmitted female progenies carrying the targeted allele were crossed with a C57BL6 male mouse expressing a Cre recombinase transgene under the control of protamine promoter to excise exon 2 and PGK-Neo cassette in sperms to produce heterozygous (+/−) CPEB4 mice. The tail genomic DNAs digested with HindIII was analyzed on a Southern blot to examine the removal of exon 2. Approximately 2.1-kb and 1-kb HindIII-excised fragments were detected from the WT and KO alleles, respectively (Figure 1B). The male littermates from the heterozygous matings were used for all of the experiments in this study. When exon 2 was deleted, a premature termination codon (PTC) was created in exon 3. Using a radioactive probe against the exon 1 region, we found the presence of CPEB4 RNA in the KO brain (Figure 1C). The reverse transcription-coupled qPCR (RT-qPCR) assay with a set of primers spanning exon 7 and exon 8 region determined approximately 30% of CPEB4 RNA remained in the KO brain (Figure 1D), suggesting that the premature stop codon in the truncated CPEB4 transcript did not efficiently trigger non-sense-mediated RNA decay. Moreover, RT-PCR detected a shorter CPEB4 isoform. Sequencing revealed this isoform did not contain exon 3 (51-bp) and exon 4 (24-bp). The deletion of exon 2 also generated a PTC in exon 6 of this isoform (Figure 1E). Because the exon 2-deleted CPEB4 transcript was not completely decayed, we next examined whether any partial CPEB4 protein encoded from exon 1 (375 a.a) remained in the KO tissues. We used the polyclonal antibody affinity-purified against the 375 a.a. of CPEB4 for immunoblotting and found no detectable truncated CPEB4 in the KO tissues (Figure 2A). The immunohistochemical staining of coronal brain sections revealed that CPEB4 was widely expressed in the brain, particularly enriched in the hippocampus and also detected in the amygdala (Figure 2B, 2C). No signal was present in the KO brain. Thus, we have created a mouse line deficient in CPEB4 protein.

### CPEB4 KO Mice Showed Normal Anxiety and Motor Coordination

**Figure 3:**
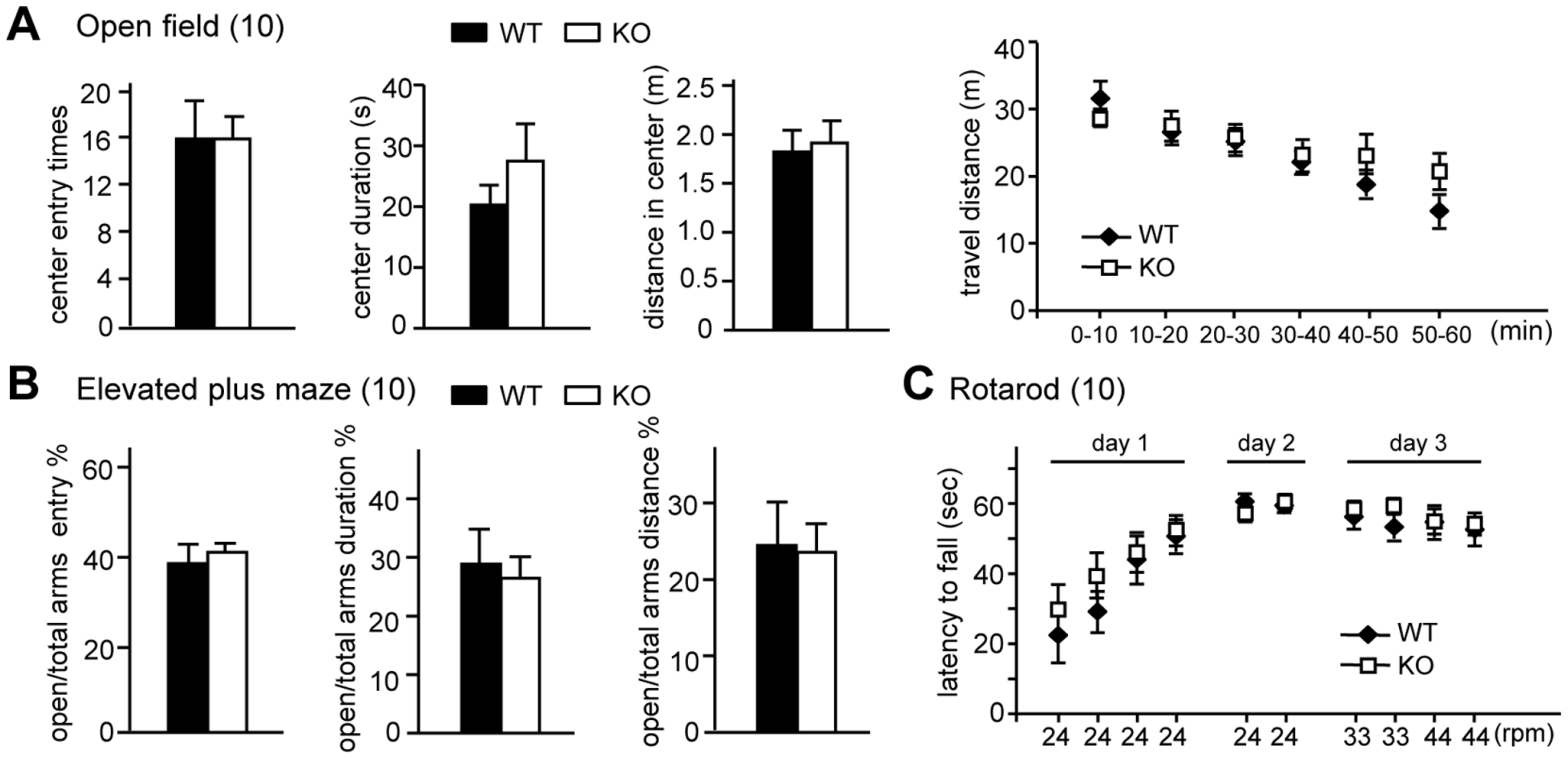
Behavioral characterization of CPEB4 KO mice. The anxiety level and exploratory behavior of WT and KO mice were assessed using the (**A**) open field and (**B**) elevated plus maze (EPM) tasks. (**A**) The mice were placed in an arena and recorded for 1 h. The entry times, staying duration and traveling distance in the center during the first 10 min movement were analyzed and presented as bar graphs. The total traveling distance in the arena in every 10 min for 1 h was shown. (**B**) The percentage of entry and staying duration into the open arms vs. the total arms in the 5 min recording of movement in the EPM was analyzed. (**C**) Motor coordination ability was assessed by the rotarod test. The latency in which the mouse was able to stay on the rotating rod of various speeds was analyzed. Numbers in parentheses denote the number of mice in each group used for the study. All of the data were expressed as the mean ± SEM.

The following behavior studies were blindly conducted to the genotypes using 2–3-month-old male WT and KO littermates. Breeding of heterozygous mice produced offspring in the expected Mendelian distribution. The body weight (WT: 22.63±0.44 g; KO: 22.02±0.42 g, n = 20) between the two groups of mice was similar. Although CPEB4 is involved in regulating meiotic and mitotic cell cycle progression and neuronal viability [8], [13], [16], we found lack of CPEB4 did not affect the growth and survival of the mice. Both male and female knockouts were fertile. Anxiety-like responses and exploratory behaviors were studied in the open field and EPM. Normally, mice fear an open environment and tend to avoid the center of the field or the elevated open arm. The extent of anxiety was determined by counting the number of entries and the duration of stay by the test mouse into the center zone in the open field (Figure 3A) or into the open arm in the EPM (Figure 3B). The KO mice showed comparable anxiety with their WT littermates in both tests. Moreover, the motor coordination of CPEB4 null mice also appeared normal because they could stay on the rotating rod as long as the WT mice did after training (Figure 3C).

### CPEB4 KO Mice Displayed Normal Hippocampus-Related Learning and Memory

**Figure 4:**
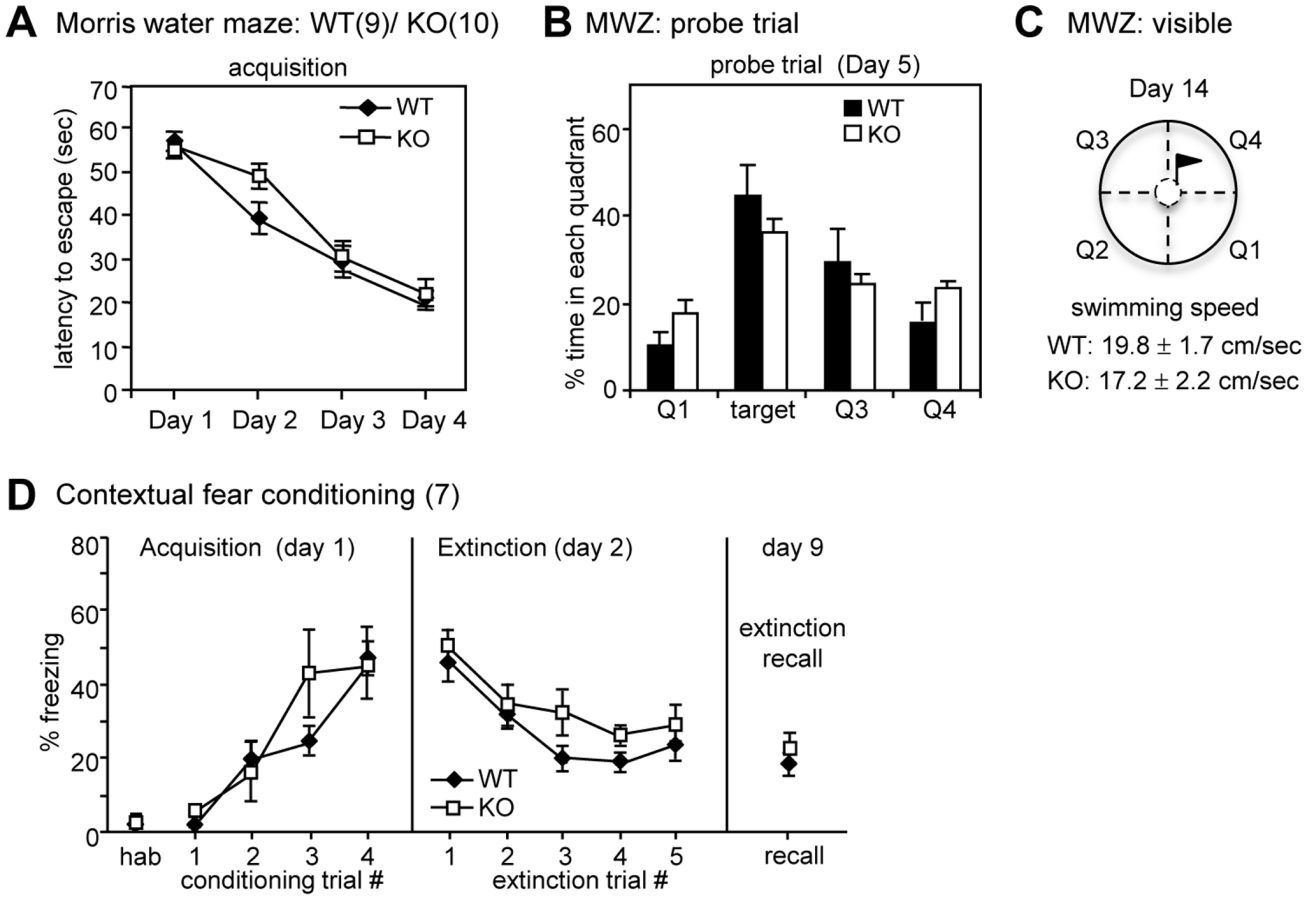
CPEB4 KO mice performed normally in the Morris water maze (MWZ) and contextual fear conditioning tests. (**A**) The WT and KO mice normally responded in the hidden platform version of MWZ within 4 trials/day for 4 consecutive days. (**B**) In the probe trial, the platform was removed, and the percentage of time that the mouse spent on navigating in each quadrant was analyzed. Both of the groups of mice searched closer to the target quadrant where the platform had been previously placed. (**C**) The motor ability and visual acuity of the mice were assessed by the swimming speed in the cued version of MWZ, in which the platform was indicated by a visible flag. (**D**) Contextual fear conditioning. The freezing response during habituation (hab) and acquisition was analyzed for 2 min per trial. A foot shock (2-sec 0.5 mA) was given at the end of habituation and the first three conditioning trials. During the extinction phase, the mouse was placed in the chamber for 30 min without reinforcing the shock. The freezing level was analyzed every 6 min for 5 segments. A week after the extinction training, the consolidated extinction memory was recalled by monitoring the freezing behavior for 6 min in the original chamber. Numbers in parentheses denote the number of mice in each group used for the study. All of the data were expressed as the mean ± SEM.

We next used the Morris water maze (MWZ) and contextual fear conditioning tests to examine hippocampus-dependent memory. During spatial acquisition in the MWZ, the mice learned to locate a hidden platform using visual cues around the maze. Both the WT and KO mice learned where the platform was positioned as evidenced by the decreasing latencies over the 4-day training period (*F*_3, 51_ = 52.919, *P*<0.001, two-way repeated measures ANOVA); however, no difference in spatial learning between groups was observed (*F*_1, 17_ = 1.606, *P = *0.206, two-way repeated measures ANOVA) (Figure 4A). During the probe trial, the hidden platform was removed and the percentage of time that the mice spent in the target quadrant, where the platform was previously placed, was recorded to determine their consolidated long-term memory. Both WT and KO mice spent a significant time in the target quadrant (WT: *F*_3, 32_ = 8.032, *P*<0.001; KO: *F*_3, 36_ = 10.067, *P*<0.001, one-way ANOVA). Nevertheless, the consolidated spatial memory between WT and KO mice was comparable because both groups of mice spent similar time in the target quadrant (*F*_1,17_ = 8.673, *P = *0.263, Student’s *t*-test) (Figure 4B). Finally, using the visible platform, we ensured that the swimming ability and visual acuity of the KO mice were normal (Figure 4C).

During contextual fear conditioning, the mice learned to express a fear response (i.e., freezing) when faced with a conditioned stimulus (CS, the chamber environment), which was previously paired with a noxious unconditioned stimulus (US, electrical foot shock). After that, if the mice were exposed only to the CS without US pairing, then previously acquired fear responses would gradually decline, a process known as extinction. A chamber with an electrified floor grid and a video camera (Clever Sys FreezeScan) was used to measure the freezing response in mice. The freezing levels in WT and KO mice were low during habituation, increased significantly after CS-US paired trainings (i.e., acquisition, *F*_4, 48_ = 1.592, *P*<0.05, two-way repeated-measures ANOVA) and were significantly reduced during extinction (*F*_4, 48_ = 8.538, *P*<0.01, two-way repeated-measures ANOVA), indicating that both of the groups of mice could perform associated learning tasks (Figure 4D). Nonetheless, the KO group learned similarly with their WT littermates during acquisition and extinction (*F*_1, 12_ = 0.255, *P = *0.627 and *F*_1, 12_ = 3.4796, *P = *0.064, respectively, two-way repeated-measures ANOVA). The consolidated long-term fear memory and extinction memory, as determined one day after acquisition (i.e., the freezing response in the first extinction trial) and recalled at 7 days after extinction, respectively, were normal in the KO mice (Figure 4D).

### Normal LTD and LTP in the CPEB4-Deleted Hippocampus

**Figure 5:**
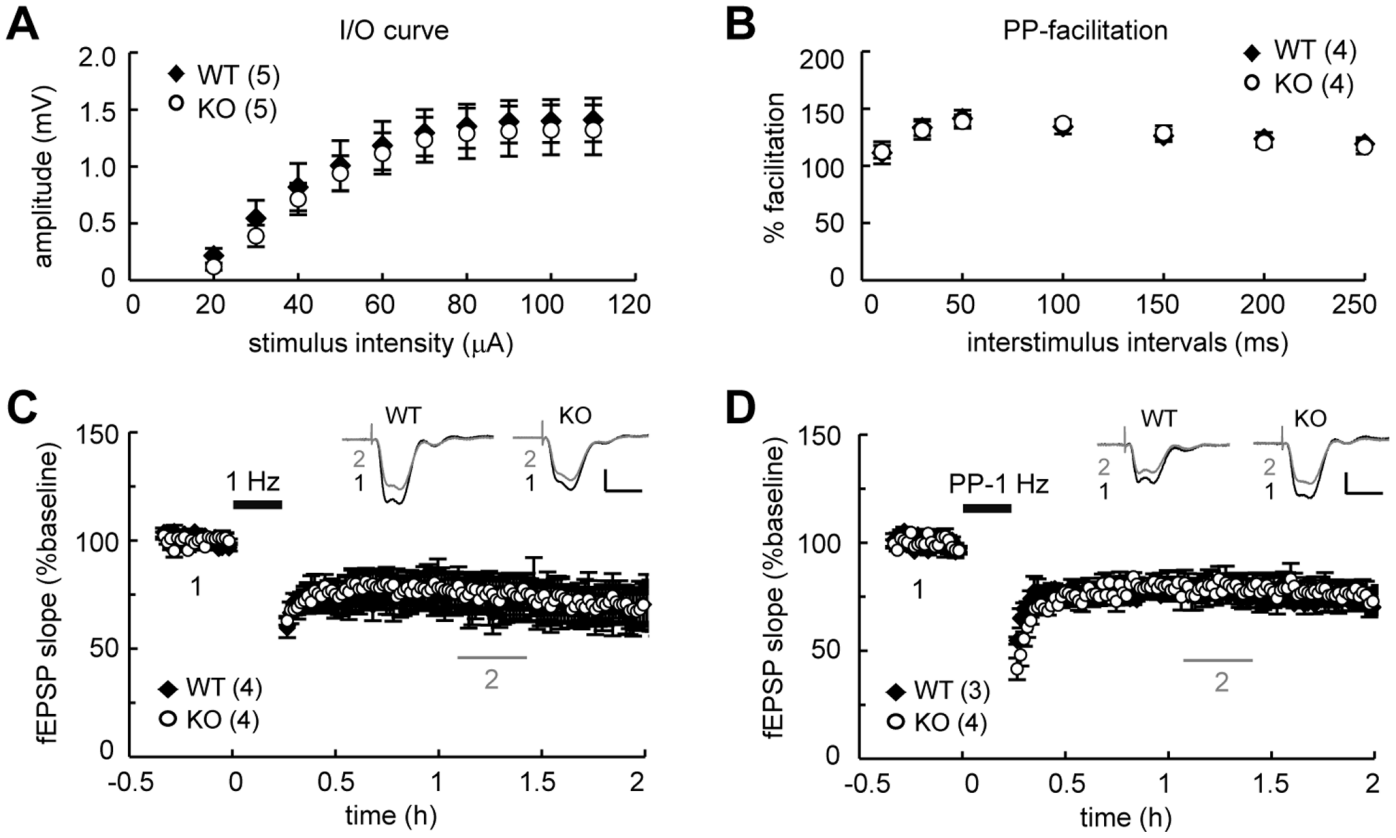
Genetic ablation of CPEB4 did not affect LTD in the SC pathway of juvenile (3-4-week-old) hippocampal slices. Basal synaptic transmission: (**A**) input-output (I–O) curve and (**B**) paired-pulse (PP) facilitation did not show a significant difference between the WT and KO mice (*P = *0.97 and 1.00, respectively, two-way ANOVA). (**C**) Normal low frequency stimulus (LFS, 1 Hz, 900 pulses)-induced LTD (*P* = 0.32, Student’s *t*-test, at 50–70 min after stimulation) and (**D**) PP-LFS-elicited LTD in the KO hippocampal slices (*P* = 0.11, Student’s *t*-test, at 50–70 min after stimulation). Numbers in parentheses represent the number of recorded slices isolated from 2–3 male mice. All of the data were expressed as the mean ± SEM. Inset: traces represent baseline (black line, 1) and 50–70 min after stimulation (gray line, 2). Calibration: 0.5 mV, 20 msec.

**Figure 6:**
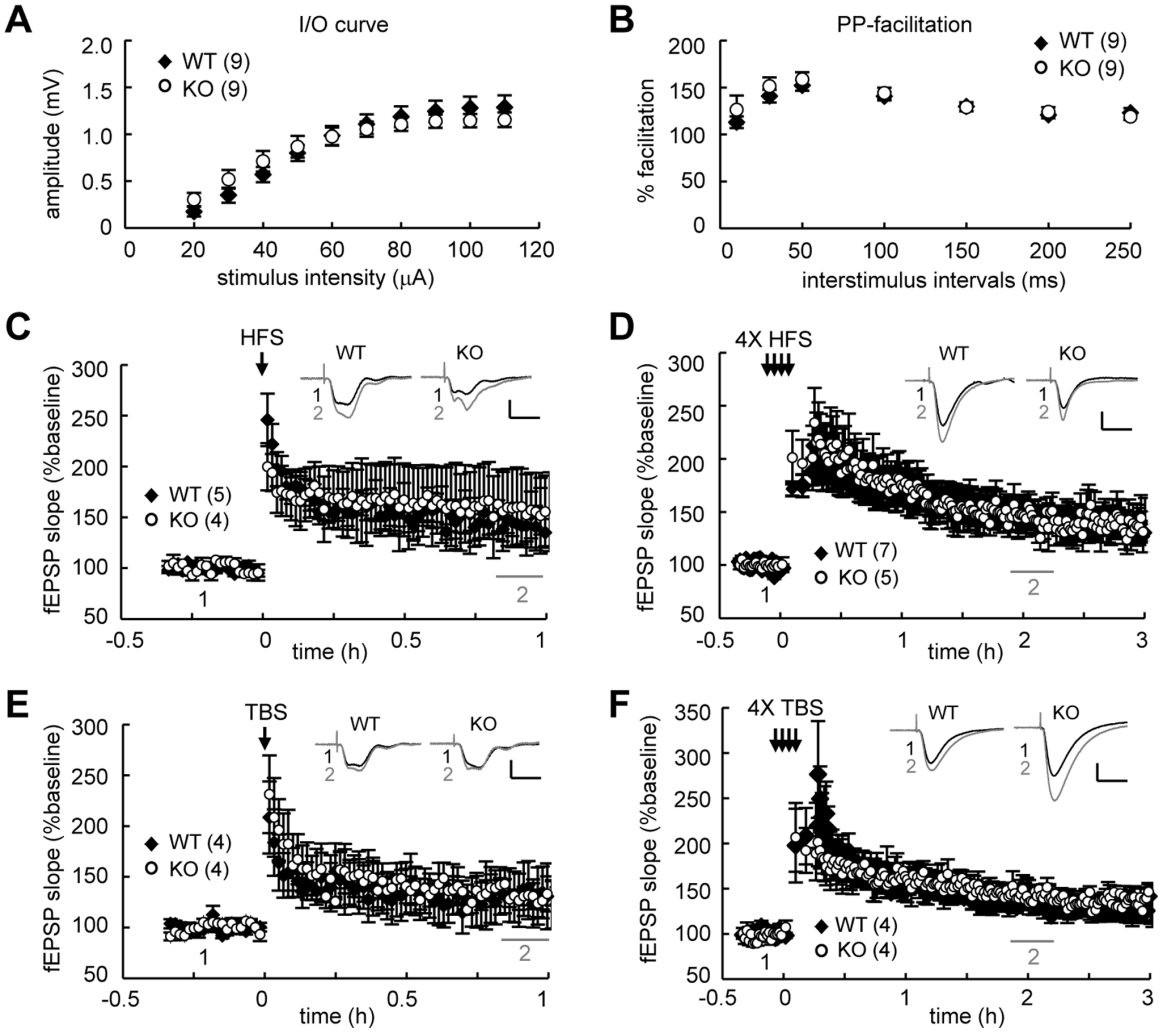
Genetic deletion of CPEB4 did not affect LTP in the SC pathway of adult (9-12-week-old) hippocampal slices. Basal synaptic transmission, (**A**) input-output (I–O) curve and (**B**) paired-pulse (PP) facilitation were normal in the KO slices (*P = *0.63 and 0.91, respectively, two-way ANOVA). No significant difference was observed between the WT and KO slices in LTP evoked by (**C**) one train (1X) of high frequency stimulation (HFS, *P* = 0.27 at 50–60 min after stimulation), (**D**) four trains (4X) of HFS (*P* = 0.69 at 100–120 min after stimulation), (**E**) 1X theta burst stimulation (TBS, *P* = 0.98 at 50–60 min after stimulation) and (**F**) 4X TBS (*P* = 0.11 at 100–120 min after stimulation). Statistics from (**C**) to (**F**) were analyzed using Student’s *t*-test. Numbers in parentheses represent the number of recorded slices isolated from 2–4 male mice. Inset: traces represent baseline (black line, 1) and the indicated time after stimulation (gray line, 2). Calibration: 0.5 mV, 20 msec.

To examine whether specific forms of synaptic plasticity were altered in the SC-CA1 pathway of the KO hippocampus, we used hippocampal slices isolated from juvenile and adult mice for long-term depression (LTD) and long-term potentiation (LTP) studies, respectively. The basic synaptic responses, including the input-output relationship (Figures 5A, 6A) and paired-pulse facilitation (Figures 5B, 6B), did not differ between the WT and KO groups. LTD induced by low-frequency stimulation (LFS) (Figure 5C, WT: 73.16±9.77%; KO: 75.51±7.10%, *P* = 0.32 at 50–70 min after stimulation), and PP-LFS was normal in young KO hippocampal slices (Figure 5D, WT: 76.76±1.68%; KO: 78.80±6.23%, *P* = 0.11 at 50–70 min after stimulation). Adult KO hippocampal slices were analyzed for potential deficits in LTP evoked by one or four trains of high frequency stimulation (1X, 4X HFS) or theta-burst stimulation (1X or 4X TBS) [20], [21]. All forms of LTP were normal in the KO slices: 1X HFS-LTP (Figure 6C, WT: 144.97±17.19%, KO: 157.31±37.64%, *P* = 0.27 at 50–60 min after stimulation); 4X HFS-LTP (Figure 6D, WT: 146.72±9.26%, KO: 145.24±15.84%, *P* = 0.69 at 100–120 min after stimulation); 1X TBS-LTP (Figure 6E, WT: 131.87±25.71%; KO, 131.66±10.46%, *P* = 0.98 at 50–60 min after stimulation); 4X TBS-LTP (Figure 6F, WT: 136.21±10.13%, KO: 141.30±10.83%, *P* = 0.11 at 100–120 min after stimulation). Thus, ablation of the *cpeb4* gene did not affect the aforementioned forms of synaptic plasticity in the SC-CA1 pathway.

### The Dendritic Spines in CPEB4 KO Neurons Were Slightly Elongated

**Figure 7:**
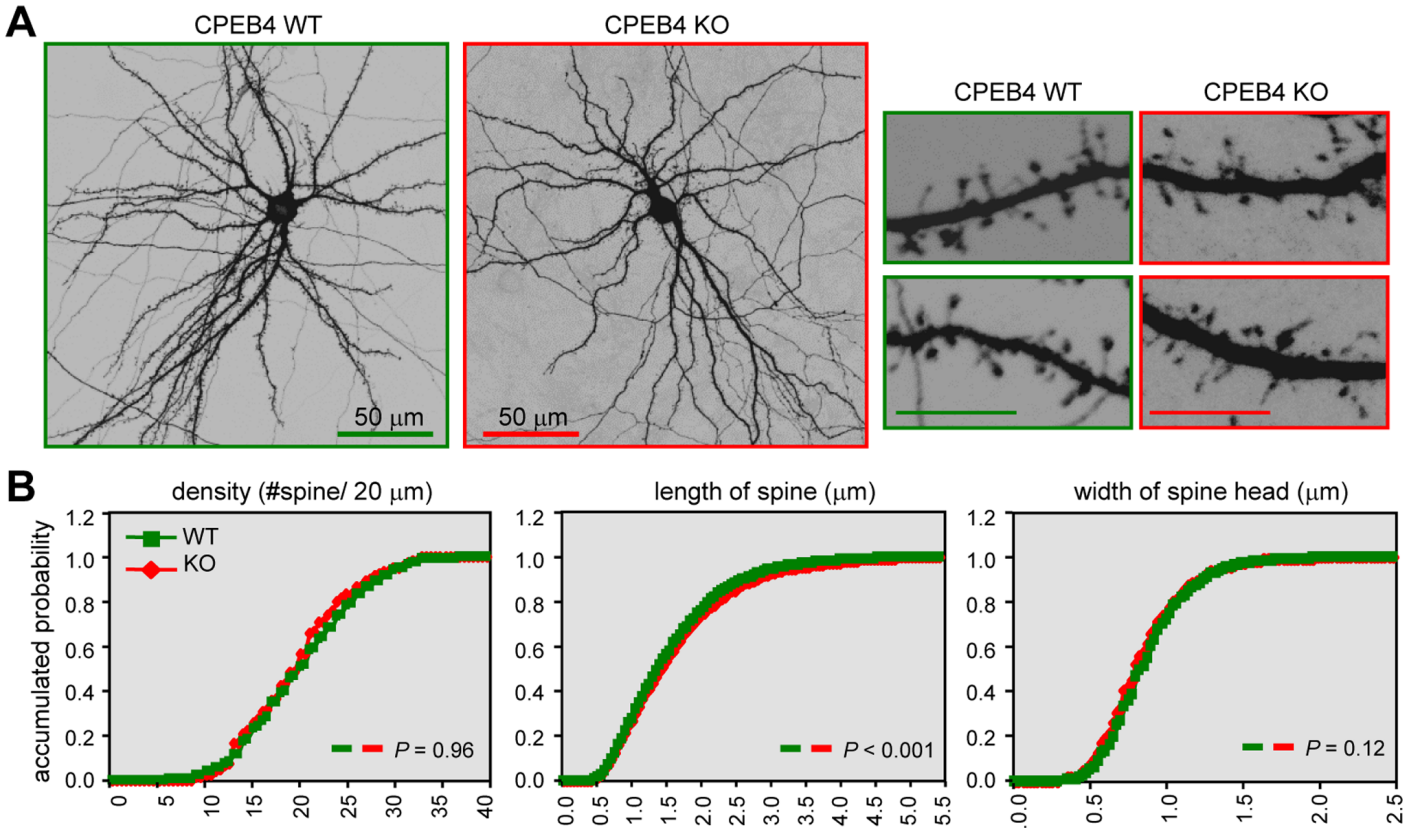
The dendritic spine length was slightly increased in CPEB4 KO neurons. (**A**) The WT and KO neurons were transfected with the GFP plasmid on DIV14 and fixed on DIV18 for GFP immunostaining. Representative images of the whole neurons and dendritic spine area are shown. Scales, 10 µm unless denoted. (**B**) Quantification analysis of dendritic spine morphology. Approximately 30 pyramidal neurons in each group (WT, green; KO, red) were collected from three independent cultures and analyzed using the MetaMorph software. The cumulative probability curves of density, length and width of dendritic spines in each group (∼3000 spines) were plotted and analyzed for the statistical difference between groups using Student’s *t*-test.

Behavioral and electrophysiological studies indicated that the KO mice had no obvious abnormalities in hippocampus-related plasticity, learning and memory. Because CPEB3 null mice showed potentiated consolidated spatial memory and CPEB3-lacking neurons had enlarged dendritic spines [22], we expected no or very subtle change in the spine morphology of CPEB4 KO neurons. The cortical/hippocampal tissues isolated from WT and KO E18 embryos were used for neuronal cultures. Although CPEB4 KO mice are fertile, the embryos of WT and KO genotypes were collected from heterozygous matings to ensure the neurons were isolated from embryos of the same developmental stages. The WT and KO neurons at 14 DIV were transfected with a plasmid expressing EGFP, fixed for immunostaining at 18 DIV, and the spine morphology of WT and KO pyramidal neurons was analyzed. Overall, depletion of CPEB4 did not affect gross morphology of pyramidal neurons (Figure 7A). However, when compared to WT neurons at higher magnification, the length of the spine (but not the density and width of the spine) in KO neurons was slightly but significantly increased (Figure 7B, right panel, WT, green line; KO, red line). Taken together, depletion of CPEB4 causes a subtle change in dendritic spine morphology that is not sufficient to affect electrophysiological plasticity and memory performance in the KO mice.

## Discussion

We have used Cre-loxp strategy to generate CPEB4 KO mice for physiological study of this protein *in vivo.* Although CPEB4 is widely distributed in various tissues and highly expressed in the hippocampus, the behavioral and electrophysiological assessments in this study indicate that loss of CPEB4 does not overtly affect hippocampus-dependent plasticity and memory. The only subtle difference we could identify is that the dendritic spines of CPEB4-depleted neurons are slightly lengthened. Nevertheless, this mild cellular defect seems not to affect learning and memory ability of the KO mice, at least in the standard MWZ and contextual fear conditioning tasks.

While the lack of obvious phenotypes in regard to growth, fertility, synaptic electrophysiology and behavior in the CPEB4 null mice, raises the issues about the specificity of knockdown approach in the culture systems vs. the possible compensatory mechanism using the genetic ablation strategy, we need to make numerous cautious comments before excluding the role of CPEB4 in cell cycle and memory. First, there are four CPEBs in vertebrates. CPEBs2–4 are 96% identical in the RBD region and share 45% and 87% identity with CPEB1 and their *Drosophila* homologue, Orb2, respectively, but they show no significant homology with CPEB1 and Orb2 in the N-terminal regulatory region. Even though CPEB4 is more similar to CPEB2 and CPEB3 at the amino acid sequence level, the previous studies indicate that CPEB4 is more functionally related to CPEB1. For example, CPEB1 and CPEB4 sequentially promote polyadenylation-induced translation of oocyte RNAs to drive the first (metaphase I) and second (metaphase II) meiotic divisions, respectively. However, if expression of a stable and constitutively active CPEB1 mutant to last CPEB1’s function until metaphase II, the meiotic defect caused by the knockdown of CPEB4 could be rescued [13]. Similarly, downregulation of both CPEB1 and CPEB4 in HeLa cells caused more severe defects in polyadenylation and gene expression during mitosis and accumulated more cells arrested at the G2/M phase than just knocking down one of them [8]. Thus, it is very possible that CPEB1 can partially compensate the loss of CPEB4, leading to no obvious defect in growth and fertility of the KO mice. The other possibility is that CPEB4 may affect mitotic entry in cancer cells but not in normal cells. Alternatively, it is just fundamentally difficult to compare the physiological consequence arisen from acute downregulation vs. complete depletion of a protein. Delivery of the virus expressing Cre recombinase to acutely knock out *cpeb4* gene in the hippocampi of mice carrying the *cpeb4* conditional alleles in the future study may help clarify this issue.

Because Orb2 is necessarily required for consolidating long-term memory of courtship behavior in the fly [23], we hypothesize that some, if not all, of its mammalian homologues, CPEBs2–4, may be indispensable for memory consolidation. Surprisingly, the mice ablated with *cpeb3* gene have facilitated LTD in the hippocampus and potentiated memory consolidation. Our molecular and cellular characterizations reveal that CPEB3 functions as a negative regulator to constrain the syntheses of several molecules, such as PSD95 and NMDA receptor subunits, at glutamatergic synapses and restricts the strength of consolidated memory [22]. Disruption of *cpeb1* gene in mice reduces extinction of hippocampus-dependent LTM [9] and altered synaptic electrophysiology in the SC-CA1 pathway [20]. On the contrary, using the same behavioral tests, CPEB4 null mice exhibited no obvious defects in learning and memory [9], [22]. CPEB4 possesses the same sequence-binding specificity with CPEB2 and CPEB3 *in vitro* [2] but is functionally related to CPEB1 during cell cycle [8], [13]. Although it is conceivable that elimination of CPEB4 could have resulted in compensatory mechanisms through which another CPEB substitutes for memory function normally exerted by CPEB4, it is also possible that the abundant expression of CPEB4 in the hippocampus may have other functions except learning and memory, possibly like promoting neuronal survival after cerebral ischemia [16]. Further investigation is needed to uncover, if any, the nature of potential compensatory mechanisms or other abnormalities in the CPEB4 KO mice.

## References

[1] Theis M, Si K, Kandel ER (2003) Two previously undescribed members of the mouse CPEB family of genes and their inducible expression in the principal cell layers of the hippocampus. Proc Natl Acad Sci U S A 100: 9602–9607. 12871996 10.1073/pnas.1133424100 PMC170964

[2] Huang YS, Kan MC, Lin CL, Richter JD (2006) CPEB3 and CPEB4 in neurons: analysis of RNA-binding specificity and translational control of AMPA receptor GluR2 mRNA. EMBO J 25: 4865–4876. 17024188 10.1038/sj.emboj.7601322 PMC1618119

[3] Alexandrov IM, Ivshina M, Jung DY, Friedline R, Ko HJ, et al (2012) Cytoplasmic polyadenylation element binding protein deficiency stimulates PTEN and Stat3 mRNA translation and induces hepatic insulin resistance. PLoS Genet 8: e1002457. 22253608 10.1371/journal.pgen.1002457 PMC3257279

[4] Burns DM, Richter JD (2008) CPEB regulation of human cellular senescence, energy metabolism, and p53 mRNA translation. Genes Dev 22: 3449–3460. 19141477 10.1101/gad.1697808 PMC2607074

[5] Groisman I, Ivshina M, Marin V, Kennedy NJ, Davis RJ, et al (2006) Control of cellular senescence by CPEB. Genes Dev 20: 2701–2712. 17015432 10.1101/gad.1438906 PMC1578696

[6] Groisman I, Jung MY, Sarkissian M, Cao Q, Richter JD (2002) Translational control of the embryonic cell cycle. Cell 109: 473–483. 12086604 10.1016/s0092-8674(02)00733-x

[7] Tay J, Richter JD (2001) Germ cell differentiation and synaptonemal complex formation are disrupted in CPEB knockout mice. Dev Cell 1: 201–213. 11702780 10.1016/s1534-5807(01)00025-9

[8] Novoa I, Gallego J, Ferreira PG, Mendez R (2010) Mitotic cell-cycle progression is regulated by CPEB1 and CPEB4-dependent translational control. Nat Cell Biol 12: 447–456. 20364142 10.1038/ncb2046

[9] Berger-Sweeney J, Zearfoss NR, Richter JD (2006) Reduced extinction of hippocampal-dependent memories in CPEB knockout mice. Learn Mem 13: 4–7. 16452649 10.1101/lm.73706

[10] Chen PJ, Huang YS (2012) CPEB2-eEF2 interaction impedes HIF-1alpha RNA translation. EMBO J 31: 959–971. 22157746 10.1038/emboj.2011.448 PMC3280548

[11] Wang CF, Huang YS (2012) Calpain 2 activated through N-methyl-D-aspartic acid receptor signaling cleaves CPEB3 and abrogates CPEB3-repressed translation in neurons. Mol Cell Biol 32: 3321–3332. 22711986 10.1128/MCB.00296-12 PMC3434545

[12] Pavlopoulos E, Trifilieff P, Chevaleyre V, Fioriti L, Zairis S, et al (2011) Neuralized1 activates CPEB3: a function for nonproteolytic ubiquitin in synaptic plasticity and memory storage. Cell 147: 1369–1383. 22153079 10.1016/j.cell.2011.09.056 PMC3442370

[13] Igea A, Mendez R (2010) Meiosis requires a translational positive loop where CPEB1 ensues its replacement by CPEB4. EMBO J 29: 2182–2193. 20531391 10.1038/emboj.2010.111 PMC2905248

[14] Xi H, Schwartz R, Engel I, Murre C, Kersh GJ (2006) Interplay between RORgammat, Egr3, and E proteins controls proliferation in response to pre-TCR signals. Immunity 24: 813–826. 16782036 10.1016/j.immuni.2006.03.023

[15] Ortiz-Zapater E, Pineda D, Martinez-Bosch N, Fernandez-Miranda G, Iglesias M, et al (2012) Key contribution of CPEB4-mediated translational control to cancer progression. Nat Med 18: 83–90. 10.1038/nm.2540 22138752

[16] Kan MC, Oruganty-Das A, Cooper-Morgan A, Jin G, Swanger SA, et al (2010) CPEB4 is a cell survival protein retained in the nucleus upon ischemia or endoplasmic reticulum calcium depletion. Mol Cell Biol 30: 5658–5671. 20937770 10.1128/MCB.00716-10 PMC3004280

[17] Le Fur N, Kelsall SR, Mintz B (1996) Base substitution at different alternative splice donor sites of the tyrosinase gene in murine albinism. Genomics 37: 245–248. 8921397 10.1006/geno.1996.0551

[18] Chao HW, Lai YT, Lu YL, Lin CL, Mai W, et al (2012) NMDAR signaling facilitates the IPO5-mediated nuclear import of CPEB3. Nucleic Acids Res 40: 8484–8498. 22730302 10.1093/nar/gks598 PMC3458550

[19] Morris R (1984) Developments of a water-maze procedure for studying spatial learning in the rat. J Neurosci Methods 11: 47–60. 6471907 10.1016/0165-0270(84)90007-4

[20] Alarcon JM, Hodgman R, Theis M, Huang YS, Kandel ER, et al (2004) Selective modulation of some forms of schaffer collateral-CA1 synaptic plasticity in mice with a disruption of the CPEB-1 gene. Learn Mem 11: 318–327. 15169862 10.1101/lm.72704 PMC419735

[21] Patterson SL, Pittenger C, Morozov A, Martin KC, Scanlin H, et al (2001) Some forms of cAMP-mediated long-lasting potentiation are associated with release of BDNF and nuclear translocation of phospho-MAP kinase. Neuron 32: 123–140. 11604144 10.1016/s0896-6273(01)00443-3

[22] Chao HW, Tsai LY, Lu YL, Lin PY, Huang WH, et al (2013) Deletion of CPEB3 Enhances Hippocampus-Dependent Memory via Increasing Expressions of PSD95 and NMDA Receptors. J Neurosci 33: 17008–17022. 24155305 10.1523/JNEUROSCI.3043-13.2013 PMC6618447

[23] Keleman K, Kruttner S, Alenius M, Dickson BJ (2007) Function of the Drosophila CPEB protein Orb2 in long-term courtship memory. Nat Neurosci 10: 1587–1593. 17965711 10.1038/nn1996

